# Virus-Like Particle Mediated CRISPR/Cas9 Delivery for Efficient and Safe Genome Editing

**DOI:** 10.3390/life10120366

**Published:** 2020-12-21

**Authors:** Pin Lyu, Luxi Wang, Baisong Lu

**Affiliations:** 1 School of Physical Education and Health, Hangzhou Normal University, Hangzhou 311121, China; pinlyu@hznu.edu.cn; 2 Department of Cancer Biology, Comprehensive Cancer Center of Wake Forest Baptist Medical Center, Winston-Salem, NC 27157, USA; luxwang@wakehealth.edu; 3 Wake Forest Institute for Regenerative Medicine, Wake Forest University Health Sciences, Winston-Salem, NC 27157, USA

**Keywords:** virus-like particle (VLP), viral capsid, gene editing, designer nuclease, delivery, RNA, ribonucleoprotein, ZFN, TALEN, CRISPR/Cas9

## Abstract

The discovery of designer nucleases has made genome editing much more efficient than before. The designer nucleases have been widely used for mechanistic studies, animal model generation and gene therapy development. However, potential off-targets and host immune responses are issues still need to be addressed for *in vivo* uses, especially clinical applications. Short term expression of the designer nucleases is necessary to reduce both risks. Currently, various delivery methods are being developed for transient expression of designer nucleases including Zinc Finger Nuclease (ZNF), Transcription Activator-Like Effector Nuclease (TALEN) and Clustered Regularly Interspaced Short Palindromic Repeats/CRISPR-associated (CRISPR/Cas). Recently, virus-like particles are being used for gene editing. In this review, we will talk through commonly used genome editing nucleases, discuss gene editing delivery tools and review the latest literature using virus-like particles to deliver gene editing effectors.

## 1. An Introduction to Genome Editing Nucleases

Genome editing is a technology that enables human beings to edit the target genome and achieve the knockout and addition of specific DNA fragments within a cell or organism by designer endonucleases [1]. Early gene editing was based on homologous recombination targeting technology, which was extremely inefficient and prone to off-target effects [2]. The subsequent development of designer endonucleases changed this situation. Three major types of designer endonucleases are widely used for genome editing: Zinc Finger Nuclease (ZNF), Transcription Activator-Like Effector Nuclease (TALEN) and Clustered Regularly Interspaced Short Palindromic Repeats/CRISPR-associated (CRISPR/Cas). In this review, we will focus on the applications of these three nucleases in mammalian cells. Reviews discussing other genome editing effectors and applications in non-mammalian cells are available elsewhere [3,4,5].

ZFN was the first type of artificial endonuclease designed by Kim et al. in 1996 [6]. ZFNs consist of DNA-binding domains of zinc finger proteins and the DNA cleavage domain of endonuclease FokI. The DNA-binding domains of zinc finger proteins can recognize and bind the target sequence, then the cleavage domain of FokI creates DNA double-strand breaks at specific locations in complex genomes [6]. FokI endonuclease works as a dimer and the double-strand DNA cleavage occurs only at sites of binding of two ZFNs to the opposite DNA strands. Also, ZNF was the first artificial nuclease to be tested in clinical trials to treat HIV [7]. As the first gene-editing tool, this technology allows us to learn more about genetic engineering, gene knockdown and knockin. But the disadvantages are also obvious, including relative low efficiency and laborious design.

TALENs are the second type of artificial endonucleases with similar architecture and mechanism as ZNFs: a DNA binding domain and a cleavage domain of FokI [8]. The DNA binding domains of TALENs are found in Xanthomonas bacteria. A repeat of 33–34 amino acid sequence can recognize a specific nucleotide in the target sequence [9]. This function is realized through specific DNA-binding domains by selecting a combination of repeats. TALEN has also been moved into clinical trials to treat lymphoblastic leukemia [10]. TALENs’ advantage is that they are relatively easier to design than ZFNs. But making TALEN constructs is time-consuming and for each new target, a new nuclease has to be engineered.

The Clustered Regularly Interspaced Short Palindromic Repeats (CRISPR)/CRISPR-associated (CRISPR/Cas) is a bacterial adaptive immune system [11]. It was found that a single protein (Cas9) was responsible for the endonuclease activity and that the two small RNAs, crRNA and tracrRNA, could be fused as one single guide RNA [12]. This observation made it possible to conveniently design single guide RNA to target a specific target sequence. Following single guide RNA optimization and Cas9 nuclear import enhancement, the system was rapidly used in genome editing in eukaryotic cells [13,14,15,16]. Unlike ZNFs and TALENs that a new nuclease has to be engineered for each target sequence, the target sequence specificity of CRISPR/Cas is determined by single-guide RNA which is easier to design and generate. This simplicity makes CRISPR/Cas the most popular gene editing tool and CRISPR/Cas has been widely used in a series of research fields such as animal model generation [17], crop improvement [18], microbial genome editing [19], gene expression regulation [20,21,22], DNA and RNA labeling [23,24] and gene therapy [25,26,27]. Many CRISPR-based gene therapy clinical trials have been registered on ClinicalTrials.gov. The advantages of CRISPR/Cas system are high efficiency, multiplexed editing and easy to prepare. The critical disadvantages are off-target effects and requiring a protospacer adjacent motif (PAM) for the target sequence. To circumvent the PAM limitation, Cas9 mutants with altered PAM specificities [28], broad PAM compatibility [29] or nearly without PAM restrictions [30] are developed.

The safety of designer nucleases is an important issue when using them for the treatment of human diseases. One major concern of gene editing in clinical use is the high propensity for off-target effects [2,31]. Off-targets are positively correlated to designer nuclease expression levels and expression duration [32,33,34,35]. Thus, delivery methods can increase or decrease the generation of off-targets. Currently, designer nucleases are often delivered by plasmid DNA transfection or various viral vectors such as lentiviral vectors (LVs) and adeno-associated virus-derived vectors (AAVs). These delivery methods have the advantage of high delivery efficiency. However, they usually mediate sustained or high-level nuclease expression, which will increase the possibility of off-target effects and immune responses. In addition, LVs integrate the transgene into the genome of target cells and AAVs are prone to integrate the vector DNA into the target site [36]. The safety of designer nucleases can be increased by decreasing the expression duration through delivering designer nuclease proteins or RNPs by electroporation [32], cell-penetrating peptides [37], cationic lipid [38] and gold nanoparticles [39]. However, these physical and chemical delivery methods may cause damage to the cells, be inefficient or incompatible with *in vivo* applications [40].

Recently a new type of delivery vehicle, virus-like particles (VLPs), has been developed for gene editing (see studies listed in Table 1 and Table 2). These particles have the majority of the normal viral vector components, such as the envelope and capsids but not the virus’ genome. The main function of capsids is to encapsidate the viral genome within virions in one host, to transport it and subsequently release it inside another host cell [41]. Most virus capsid structures are helical or icosahedral [42,43]. Nowadays, scientists are using VLPs as delivery tools to combine the high infection efficiencies of viral vectors and the transient feature of mRNA, protein and RNP delivery. These delivery tools package mRNAs, proteins or RNPs into viral capsids for efficient and safe genome editing. The most widely used capsids are lentiviral capsids [44].

In this review, we will introduce lentiviral capsid proteins and their functions. Then we focus on how to use viral capsid proteins to package designer nucleases in mRNA, protein or RNP form and use the resulting VLPs to do genome editing. Many methods were developed to generate VLPs for safe and efficient genome editing (Table 1 and Table 2). We will first describe methods for VLP mediated nuclease mRNA delivery, then those for VLP mediated nuclease protein or RNP delivery. For VLP mediated protein or RNP delivery, two strategies, the fusion strategy and the aptamer and aptamer-binding protein interaction strategy, will be introduced.

## 2. Lentiviral Capsid Proteins and Their Functions

A lentiviral particle contains two copies of the lentiviral RNA genome, each about 9200 nucleotides in length [58] and a capsid consisting of about 5000 Gag (Group-specific antigen) precursor proteins [59,60]. The packaging of the viral genome in the capsid depends on the interaction between the Ψ packaging signal in the viral RNA and the nucleocapsid (NC) protein. In the host cells, lentiviruses synthesize cDNA with viral RNA as a template, make double-stranded DNA with cDNA as a template and integrate the double-stranded DNA into the host genome to achieve persistent expression [61].

**Figure 1:**
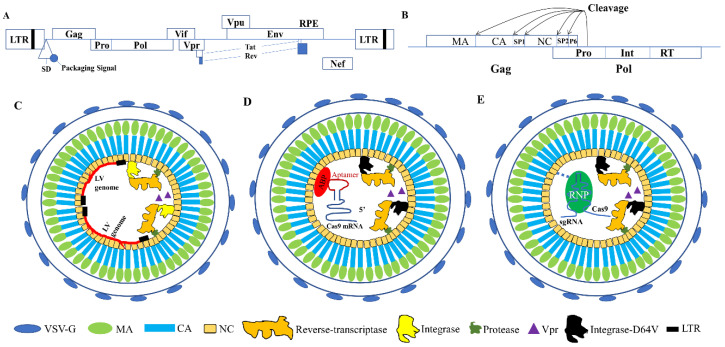
Lentiviral vector and virus-like particles. (**A**) Structure of the HIV-1 genome and its encoded proteins. (**B**) Proteins encoded by Gag and Pol genes. The cleavage sites for the protease (Pro) encoded by the Pol gene are indicated by arrows. (**C**) Diagram illustrating normal lentiviral vector. The presence of long terminal repeat (LTR) in the RNA genome, reverse transcriptase and integrase make it possible to integrate the DNA into the host cell genome. (**D**) Diagram illustrating mRNA-delivering VLPs. The mRNA does not contain LTR so that reverse transcription cannot happen. The mRNA can only serve as the template for translation. (**E**) Diagram illustrating protein- or RNP-delivering VLPs. The RNA, if present, does not contain LTR so that reverse transcription cannot happen.

Currently, the most widely used lentiviral vectors are modified from the human immunodeficiency virus type 1 (HIV-1) pre-viral genome (Figure 1A–C) [62]. HIV-1 virus belongs to retrovirus and is spherical with a diameter of 80–130 nm. Its core consists of two single-stranded positive-stranded RNAs, reverse transcriptase, integrase and protease [63]. Outside the core is the viral capsid, which is mainly composed of capsid glycoprotein. The outermost layer is the envelope and the glycoprotein on the envelope determines the host cells that can be infected. Among the nine genes of the HIV-1 pre-viral genome (Gag, Pol, env, Vif, Vpr, Vpu, Nef, rev and tat), we will focus on the products of Gag and Pol, which are most relevant to the topics of this review.

The Gag gene encodes the protein precursor p55, which is hydrolyzed by a protease (encoded by Pol) to produce endomembrane matrix protein (MA, p17), capsid protein (CA, p24) and nucleocapsid protein (NC, p7), as well as other peptides (SP1, SP2 and p6) [59]. MA has a domain that is required for the transport of Gag polyprotein to the plasma membrane and a myristoylation site that associates Gag to the plasma membrane [64]. CA contains residues that form critical Gag-Gag interactions. It is the building unit of the capsids and is involved in many processes during HIV-1 infection, including reverse transcription, nuclear entry and integration of viral DNA into host cell chromatin [65]. NC is required for viral genomic RNA packaging as well as non-specific interactions with RNA [66]. The NC protein contains two zinc finger CysCysHisCys motifs, each binding to a zinc ion [67]. The NC zinc finger motifs are critical for specific genomic RNA encapsidation [68] as well as virion production [69].

The Pol gene encodes reverse transcriptase, integrase and protease. These enzymes are responsible for transforming the RNA genome into cDNA, integrating the DNA into the host genome and processing Gag precursors into mature proteins [70]. In lentiviral vectors, the HIV env proteins are replaced with vesicular stomatitis virus G protein (VSV-G) for broad host range and high infection efficiency [71,72,73].

## 3. Using VLPs as Safe Gene Editing Delivery Vehicles

**Figure 2:**
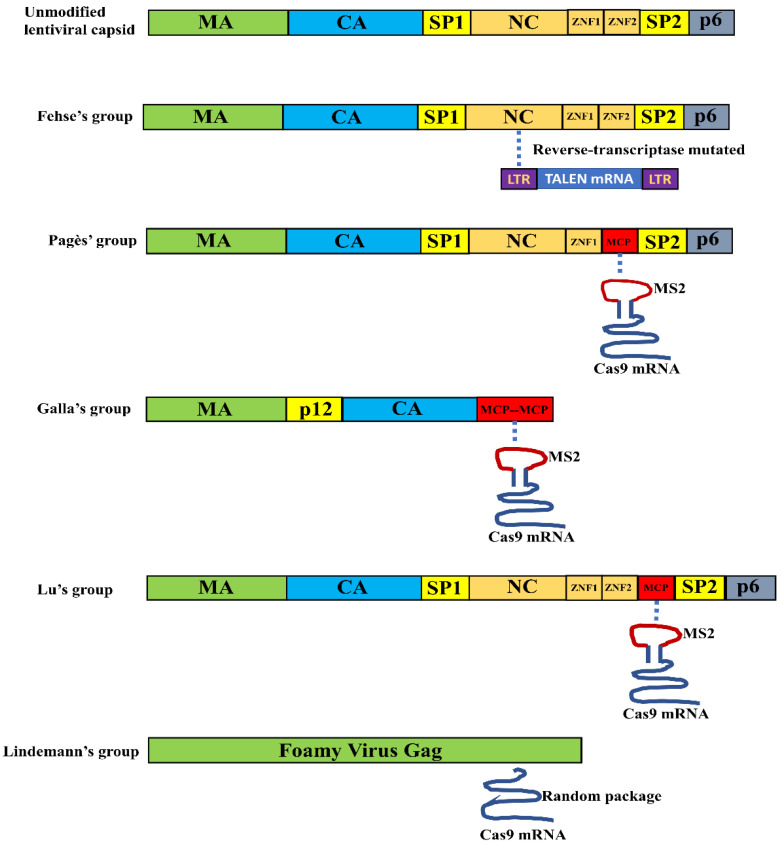
Strategies for modifying the Gag protein for mRNA delivery by VLPs. A dashed line indicates protein/RNA interactions.

**Figure 3:**
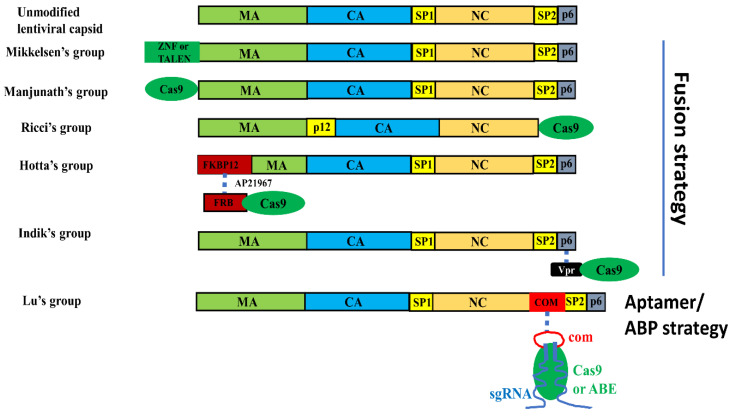
Strategies for modifying the Gag protein for endonuclease protein or RNP delivery by VLPs. A dashed line indicates non-covalent interactions. FKBP12 and FRB interaction is mediated by rapamycin analog AP21967.

The Gag polypeptide is the only viral protein required for the assembly and release of the immature virus particles, although the production of the infectious virus requires other viral proteins [44,59]. In addition, the viral genome RNA is not needed to achieve lentiviral capsid assembly and entry to the cells [74]. This feature allows us to use VLPs to deliver gene editing effectors in the form of mRNA (Figure 1D, Figure 2), proteins or RNPs (Figure 1E, Figure 3). Thus we need to figure out how these cargoes can be packaged into the capsids. We confine our discussion on works without the involvement of Cas9 DNA, thus have not included works using integration defective lentiviral vectors (IDLV) to deliver Cas9 [75,76,77]. IDLV can avoid long-term Cas9 expression but there is still a risk of random integration and the expression duration may still be longer than needed.

### 3.1. VLPs for Nuclease mRNA Delivery

Table 1 lists publications using VLPs to deliver endonuclease mRNA for gene editing and Figure 2 illustrates the mechanisms used for packaging mRNA into the particles.

The long terminal repeat (LTR) sequences of retrovirus and lentivirus contain signals for initiating reverse transcription and mediating integration into the host genome. To preserve RNA packaging but not reverse transcription and integration, Mock et al. attempted to use reverse transcriptase inactivated lentiviral vectors to deliver TALEN mRNA [45]. In this system, the packaged RNAs are not reverse transcribed or integrated. Instead, they are used as the templates for translation to transiently express the nuclease. The authors successfully used this strategy to deliver TALEN mRNA [45]. However, we failed to observe evident gene editing activity when trying to use the same method for Cas9 mRNA delivery (our unpublished data). One possible explanation is that only two copies of mRNA could be packaged by this method and the amount of Cas9 mRNA was not enough to achieve efficient gene editing.

RNA aptamer MS2 and its interacting aptamer-binding protein (ABP), MS2 coat protein (MCP) [78], has been used for RNA labeling [79,80] and protein recruitment [81,82]. Prel et al. tried to use ABP/aptamer interactions to package and deliver mRNA [46]. In this strategy, packaging no longer depends on the Ψ packaging signal near the LTR, therefore, reverse transcription and integration can be avoided. In order to package mRNA into lentiviral capsids, the authors replaced the second zinc finger domain of nucleocapsid (NC) protein with MCP which interacts with MS2 aptamer. On the other hand, they inserted 6–12 copies of MS2 aptamer to the 3′ UTR of the cargo mRNAs. They found that the mRNAs were packaged into lentiviral capsids by specific ABP/MS2 interactions and successfully delivered various mRNAs into mammalian cells, including human CD34+ and induced pluripotent stem cells [46]. They observed up to 6 copies of mRNA/particle. One issue with this method is that the particle assembly efficiency was impaired. This could be caused by the removal of NC zinc finger 2, which has been found to decrease lentiviral vector production by over 10 fold [69].

Knopp et al. used a similar strategy to package Cas9 mRNA in murine leukemia virus capsids [47]. They replaced the NC within Gag with two copies of MCP and added two copies of MS2 aptamer in the 3′ UTR of SpCas9 mRNA or various positions of sgRNA. The authors found that using this method Cas9 mRNA could be efficiently delivered into various murine and human cell lines, including human T cells and primary human fibroblasts [47]. However, sgRNA could be only functionally delivered when it was co-packaged with Cas9 mRNA. We also found that aptamer-modified sgRNA packaged alone could not be functionally delivered [55]. In the following section, we will discuss possible explanations for these observations.

Our group has also reported using lentiviral capsids to package SaCas9 mRNA [48]. We made the following modifications to improve the efficiency of mRNA packaging and delivery: (1) We inserted one copy of MCP after the second zinc finger motif of NC instead of replacing any NC domains. Consistent with the observation that NC is important for the production of retrovirus [83] and lentivirus [69], we found near 100% particle assembly efficiency in our experiments. (2) Our result indicated that when adding one copy but not multiple copies of MS2 in Cas9 3′ UTR, SaCas9 mRNA showed the best gene editing efficiency. Zalatan et al. also observed that adding more copies of aptamer decreased RNA expression [82]. (3) We included two copies of 3′ UTR sequence from human beta hemoglobin (*HBB*) in Cas9 3′ UTR to increase mRNA stability and expression [84,85]. With these modifications, we observed 50–100 copies of SaCas9 mRNA per particle and high genome editing activity [48].

Taking advantage of the foamy viruses to efficiently package non-viral cellular RNAs [86,87], Lindel et al. successfully used foamy viral capsid to package and deliver SpCas9 mRNA [49]. They observed > 80% genome editing activity and improved specificity compared with viral delivery.

It is reasonable to assume that the cells can be infected by lentiviral vectors should also be infected by VLPs, because the same pseudotyped envelopes were used. So far, VLP-mediated Cas9 mRNA delivery to mammalian cells was more successful than sgRNA delivery. Several studies have found that sgRNA packaged alone could not be functionally delivered [47,49,55]. Single guide RNA is very unstable in cells unless complexed with Cas9 protein [88]. Due to the inability to package sgRNA by viral capsids, sgRNA has to be delivered via traditional methods, such as plasmid DNA transfection and integration-defective lentiviral vectors [45,46,47,48,49].

### 3.2. Using VLPs for Protein and RNP Delivery

VLPs have been used to deliver proteins [89,90,91] for a long time. The need for transient designer nuclease expression in gene editing promoted attempts using VLPs to deliver nuclease protein or RNPs (Table 2, Figure 3). Two strategies have been used to package nucleases into viral capsids: the fusion strategy and the ABP/aptamer interaction strategy.

#### 3.2.1. VLP Mediated Nuclease Delivery Using the Fusion Strategy

Cai et al. used lentivirus-like particles to deliver ZNF and TALEN proteins [50]. ZNF or TALEN was packaged into lentiviral capsids via fusing to the N-terminus of Gag protein. The authors observed up to 24% INDEL rates on various targets in human cells. Using a similar strategy, Choi et al. successfully delivered SpCas9 proteins with VLPs [51]. Due to the intrinsic affinity between Cas9 protein and sgRNA, Cas9 RNPs can be packaged and delivered in the same particle. One issue of this fusion strategy is that the fusion impairs capsid assembly and unmodified Gag protein has to be supplemented to rescue capsid assembly [51].

Mangeot et al. reported that murine leukemia VLPs can be used to package SpCas9 RNPs [52]. They fused SpCas9 at the C-terminus of Gag (instead of the N-terminus in studies discussed earlier [50,51]) and produced VLPs with the help of unmodified packaging plasmid. When sgRNA is co-expressed with Gag-Cas9 fusion protein, Cas9 RNPs can be packaged into the capsids. The authors demonstrated up to 75% INDEL rate on various target sites. The authors did not discuss the effects of Gag-Cas9 fusion on virus-like particle assembly. However, unmodified Gag-Pol expressing DNA was included in the transfection for virus-like particle production. And particles were typically concentrated 100 times before use [52]. This delivery method has been subsequently used to deliver Cas9 RNPs targeting the Selenocysteine-tRNA[Ser]Sec gene in multiple cell types [92].

Recently, Gee et al. reported packaging Cas9 RNPs into extracellular nanovesicles for inducing exon skipping in *DMD* gene to cure Duchenne muscular dystrophy [53]. In this study, the authors fused FKBP12 to Gag and FRB to SpCas9 respectively. The specific interaction between FKBP12 and FRB in the presence of rapamycin analog AP21967 [93,94] associates Cas9 protein with HIV Gag [53]. Although the authors term these particles as “extracellular nanovesicles,” we believe the particles generated are more likely virus-like particles rather than typical extracellular vesicles without viral capsids which are appropriate to include in this review.

Vpr is a lentiviral regulatory protein with an important function in viral infection and pathogens [95]. Each lentiviral particle could have ~550 copies of Vpr [96]. Indikova et al. fused Cas9 to the N-terminus of lentiviral protein Vpr instead of Gag [54]. This strategy can efficiently package Cas9 protein into lentiviral capsids via the interaction between Vpr and p6 of Gag [97]. In this study, sgRNA was expressed from a lentiviral transfer vector co-packaged in the particles with Cas9 protein. It will be interesting to know whether editing efficiency could be different if sgRNA is packaged by sgRNA/Cas9 interaction. One concern of overexpressing Vpr is that it shows functional perturbation of cell functions through various mechanisms [95] and may be toxic to neurons [98].

#### 3.2.2. VLP Mediated Nuclease Delivery Using the ABP/Aptamer Interaction Strategy

Instead of fusing Cas9 protein to a viral protein, our group used the specific interaction between the aptamer and aptamer-binding protein (ABP) to recruit SaCas9, SpCas9 and adenine base editor (ABE) RNPs into lentiviral capsids [55,56,57,99]. For packaging SaCas9 RNPs, we inserted RNA aptamer into the sgRNA scaffold and inserted ABP into the Gag protein (after the second zinc finger domain of NC protein). In this strategy, Gag-ABP fusion protein binds to aptamer-sgRNA via the specific aptamer/ABP interaction. The intrinsic affinity between sgRNA and Cas9 protein recruits Cas9 to the complex. Cas9 protein, in turn, protects the stability of sgRNA. Our study compared four aptamer/ABP pairs (MS2/MCP [78], PP7/PCP [100], BoxB/λ p22 [101] and com/Com [102]) and three sgRNA locations (Tetraloop, ST2 loop and 3′ end) and found that replacing Tetraloop with aptamer com was the most efficient for packaging and delivering SaCas9 RNPs [55].

The same strategy can be used to package and deliver SpCas9 RNPs [56,99]. However, for SpCas9 packaging and delivery, replacing sgRNA ST2 loop with com aptamer was the most efficient way of modifying sgRNA [56]. Interestingly, SaCas9 and SpCas9 RNPs can be co-packaged in lentiviral capsids and the co-packaged RNPs are more efficient than individually packaged RNPs for multiplex gene editing [56].

In addition to Cas9 RNPs, we also successfully used the same strategy for adenine base editors (ABE) RNP delivery in human cells [57]. Our data show that the most efficient conditions for SpCas9 RNP delivery were also the best for ABE RNP delivery. Most importantly, delivering ABEs in this way eliminated guide-independent RNA off-targets, which were reported in experiments delivering ABEs by DNA transfection or viral vectors [103,104,105]. We reason that the lower ABE dosage used and the shorter term expression of ABEs contributed to these improvements.

Unlike fusing a protein to Gag impairing capsid assembly, insert ABPs into NC within Gag had little effects on capsid assembly and typically obtain 90~100% capsid assembly efficiency of normal lentiviral vectors in our experience [55,56]. For targets with open chromatin, treating cells with un-concentrated particle-containing supernatant could result in over 80% INDEL rates and <1% INDELs rates on an off-target with 1 nucleotide mismatch [55,56]. The high particle yield and gene editing activities make the ABP/aptamer interaction strategy a useful delivery method for safe and efficient gene editing. Our successful package of Cas9 protein through sgRNA/Cas9 interaction may provide a plausible explanation to Knopp et al.’s finding that sgRNA could only be functionally delivered when co-packaged with Cas9 mRNA [47]. In their setting, the gene editing activities observed could be from those sgRNAs complexed with and thus protected by Cas9 protein.

### 3.3. Advantages and Disadvantages of Using VLPs for Delivering Genome Editing Endonucleases

Here we present various methodologies that have been used to deliver Cas9 mRNA or RNPs originated from different research groups. Efficient gene editing could be achieved using un-concentrated particle-containing supernatants with diverse methods [54,55,56,57,99]. However, it is difficult to compare their relative efficiencies since different loci are targeted in different cells. A side by side comparison of the various methods may be needed to find the most suited method for a specific need.

Compared with delivering bacterially expressed genome editing effectors by electroporation or nanoparticles, VLP mediated mRNA or protein delivery has a series of advantages. First of all, the VLPs are relatively easy to produce. Plasmids are easily accessible from Addgene (see Table 1 and Table 2), labs with basic equipment can produce these VLPs for experimental use. Secondly, compared with the dosage used in electroporation experiments, typically much less amount of protein (e.g., 1/10 of those used for electroporation) is used in VLP mediated delivery [55,56,106]. This low dosage can offer a greater specificity.

Cas9 activity is inhibited by nucleosomes [107,108,109] and a low dosage of Cas9 is particularly sensitive to chromatin accessibility [110]. When target sequences are associated with heterochromatin, the low dosage feature of VLP delivery may become a disadvantage since a high dosage is needed for efficient editing. Additionally, VLP mediated RNA delivery is only efficient for Cas9 mRNA but not for sgRNA. New strategies to deliver Cas9 mRNA and sgRNA in the same particle will certainly benefit the field.

Due to the potential of inactivation by the complement system and monocytes in human circulation [111,112], retroviral and lentiviral VLP mediated genome editing effector delivery may be more suitable for *in vitro* and *ex vivo* applications than *in vivo* purposes. Expressing complement regulatory protein CD55 [113] and “do not eat me” signal CD47 [114] on the envelope protects the vectors in circulation. Besides, producing alloantigen-free particles by knocking out beta-2 microglobulin *(B2M)* gene in vector producing cells improves particle survival in circulation [115]. These measures mentioned above may help to improve the efficiency of VLP mediated *in vivo* delivery for gene editing.

Until now, no delivery method has met all needs, including safety, efficiency, easy production and low cost. The VLP gene editing delivery systems provide useful alternatives to the currently available delivery methods, such as plasmid DNA transfection, viral vectors, RNP electroporation and various nanoparticles.

## Figures and Tables

**Table 1:** Virus-like particle (VLP) mediated RNA delivery.

Virus Type	Capsid Modification	RNA Package	Copy Number	Addgene Plasmids	Reference
LV	Not modified	TALEN mRNA	2 copies	LeGO-iG2-wPRE-pA (60489)	[45]
LV	MCP replaced the second zinc finger domain of NC	SpCas9 mRNA	~6 copies	Not available	[46]
Murine Leukemia Virus	Two copies of MCP replaced NC	SpCas9 mRNA and sgRNA	Not available	Not available	[47]
LV	MCP inserted after the second zinc finger domain of NC	SaCas9 mRNA	50~100 copies	pSaCas9-1xms2-2x3′UTR (122946)	[48]
				pSaCas9-1xPP7-2x3′UTR(122947)	
				psPAX2-D64V-NC-PP7(122945)	
				psPAX2-D64V-NC-MS2(122944)	
Foamy Viruses	Not modified	SpCas9 mRNA	60 copies	Not available	[49]

**Table 2:** VLP mediated nuclease protein and RNP delivery.

Capsid Type	Mechanism of Nuclease Recruitment	Editing Effectors Delivered	Addgene Plasmids	Reference
LV	Fusing editing effector to the N-terminus of Gag	ZNF and TALEN	Not available	[50]
LV	Fusing Cas9 protein to the N-terminus of Gag	SpCas9	Not available	[51]
MLV	Fusing Cas9 to the C-terminus of MLV Gag	SpCas9	BIC-Gag-CAS9(119942)	[52]
LV	Fusing FKBP12 to Gag, fusing FRB to SpCas9. FKBP12/AP21967/FRB interaction brings SpCas9 to Gag	SpCas9	pHLS-EF1a-FRB-SpCas9-A(138477)	[53]
			pHLS-EF1a-FKBP12-Gag(HIV)(138476)	
LV	Fusing Cas9 to the C-terminus of Vpr	SpCas9	Not available	[54]
LV	Forming a three-component complex: Com-NC/aptamer-sgRNA/Cas9 protein.	SaCas9	pSaCas9-sgRNA-Tetra-com- vector(131227)	[55]
			psPAX2-D64V-NC-COM(131226)	
		SpCas9	pSpCas9-3′UTR-ST2-com-vector(136269)	[56]
		ABE	pSpCas9-ABE-3′UTR-sgRNA-ST2-com- vector(136270)	[57]

## Abbreviations

DNA	Deoxyribonucleic Acid
cDNA	complementary DNA
RNA	Ribonucleic Acid
gRNA	guide RNA
sgRNA	single guide RNA
ZFN	Zinc Finger Endonuclease
TALEN	Transcription Activator-Like Effector Nuclease
CRISPR/Cas	Clustered Regularly Interspaced Short Palindromic Repeats/CRISPR-associated/CRISPR-associated
LV	Lentiviral Vector
AAV	Adeno-Associated virus-derived Vector
HIV-1	Human Immunodeficiency Virus type 1
Gag	Group-specific antigen
MA	Matrix protein
CA	Capsid protein
NC	Nucleocapsid protein
RNP	Ribonucleoprotein
INDEL	Insertion and Deletion
VLP	Virus-like particle

## References

[1] Woolf T.M. (1998). Therapeutic repair of mutated nucleic acid sequences. Nat. Biotechnol..

[2] Fu Y., Foden J.A., Khayter C., Maeder M.L., Reyon D., Joung J.K., Sander J.D. (2013). High-frequency off-target mutagenesis induced by crispr-cas nucleases in human cells. Nat. Biotechnol..

[3] Khalil A.M. (2020). The genome editing revolution: Review. J. Genet. Eng. Biotechnol..

[4] Yang Z., Blenner M. (2020). Genome editing systems across yeast species. Curr. Opin. Biotechnol..

[5] Pramanik D., Shelake R.M., Kim M.J., Kim J.Y. (2020). Crispr-mediated engineering across the central dogma in plant biology for basic research and crop improvement. Mol. Plant.

[6] Kim Y.G., Cha J., Chandrasegaran S. (1996). Hybrid restriction enzymes: Zinc finger fusions to fok i cleavage domain. Proc. Natl. Acad. Sci. USA.

[7] Tebas P., Stein D., Tang W.W., Frank I., Wang S.Q., Lee G., Spratt S.K., Surosky R.T., Giedlin M.A., Nichol G. (2014). Gene editing of ccr5 in autologous cd4 t cells of persons infected with hiv. N. Engl. J. Med..

[8] Li T., Huang S., Jiang W.Z., Wright D., Spalding M.H., Weeks D.P., Yang B. (2011). Tal nucleases (talns): Hybrid proteins composed of tal effectors and foki DNA-cleavage domain. Nucleic Acids Res..

[9] Moscou M.J., Bogdanove A.J. (2009). A simple cipher governs DNA recognition by tal effectors. Science.

[10] Qasim W., Zhan H., Samarasinghe S., Adams S., Amrolia P., Stafford S., Butler K., Rivat C., Wright G., Somana K. (2017). Molecular remission of infant b-all after infusion of universal talen gene-edited car t cells. Sci. Transl. Med..

[11] Barrangou R., Fremaux C., Deveau H., Richards M., Boyaval P., Moineau S., Romero D.A., Horvath P. (2007). Crispr provides acquired resistance against viruses in prokaryotes. Science.

[12] Jinek M., Chylinski K., Fonfara I., Hauer M., Doudna J.A., Charpentier E. (2012). A programmable dual-rna-guided DNA endonuclease in adaptive bacterial immunity. Science.

[13] Cong L., Ran F.A., Cox D., Lin S., Barretto R., Habib N., Hsu P.D., Wu X., Jiang W., Marraffini L.A. (2013). Multiplex genome engineering using crispr/cas systems. Science.

[14] Cho S.W., Kim S., Kim J.M., Kim J.S. (2013). Targeted genome engineering in human cells with the cas9 rna-guided endonuclease. Nat. Biotechnol..

[15] Mali P., Yang L., Esvelt K.M., Aach J., Guell M., DiCarlo J.E., Norville J.E., Church G.M. (2013). Rna-guided human genome engineering via cas9. Science.

[16] Jinek M., East A., Cheng A., Lin S., Ma E., Doudna J. (2013). Rna-programmed genome editing in human cells. Elife.

[17] Wang H., Yang H., Shivalila C.S., Dawlaty M.M., Cheng A.W., Zhang F., Jaenisch R. (2013). One-step generation of mice carrying mutations in multiple genes by crispr/cas-mediated genome engineering. Cell.

[18] Bortesi L., Fischer R. (2015). The crispr/cas9 system for plant genome editing and beyond. Biotechnol. Adv..

[19] Zhang Z.T., Jiménez-Bonilla P., Seo S.O., Lu T., Jin Y.S., Blaschek H.P., Wang Y. (2018). Bacterial genome editing with crispr-cas9: Taking clostridium beijerinckii as an example. Methods Mol. Biol..

[20] Qi L.S., Larson M.H., Gilbert L.A., Doudna J.A., Weissman J.S., Arkin A.P., Lim W.A. (2013). Repurposing crispr as an rna-guided platform for sequence-specific control of gene expression. Cell.

[21] Gilbert L.A., Larson M.H., Morsut L., Liu Z., Brar G.A., Torres S.E., Stern-Ginossar N., Brandman O., Whitehead E.H., Doudna J.A. (2013). Crispr-mediated modular rna-guided regulation of transcription in eukaryotes. Cell.

[22] Bikard D., Jiang W., Samai P., Hochschild A., Zhang F., Marraffini L.A. (2013). Programmable repression and activation of bacterial gene expression using an engineered crispr-cas system. Nucleic Acids Res..

[23] Nelles D.A., Fang M.Y., O’Connell M.R., Xu J.L., Markmiller S.J., Doudna J.A., Yeo G.W. (2016). Programmable rna tracking in live cells with crispr/cas9. Cell.

[24] Chen B., Gilbert L.A., Cimini B.A., Schnitzbauer J., Zhang W., Li G.W., Park J., Blackburn E.H., Weissman J.S., Qi L.S. (2013). Dynamic imaging of genomic loci in living human cells by an optimized crispr/cas system. Cell.

[25] Nelson C.E., Hakim C.H., Ousterout D.G., Thakore P.I., Moreb E.A., Castellanos Rivera R.M., Madhavan S., Pan X., Ran F.A., Yan W.X. (2016). *In vivo* genome editing improves muscle function in a mouse model of duchenne muscular dystrophy. Science.

[26] Tabebordbar M., Zhu K., Cheng J.K.W., Chew W.L., Widrick J.J., Yan W.X., Maesner C., Wu E.Y., Xiao R., Ran F.A. (2016). *In vivo* gene editing in dystrophic mouse muscle and muscle stem cells. Science.

[27] Long C., Amoasii L., Mireault A.A., McAnally J.R., Li H., Sanchez-Ortiz E., Bhattacharyya S., Shelton J.M., Bassel-Duby R., Olson E.N. (2016). Postnatal genome editing partially restores dystrophin expression in a mouse model of muscular dystrophy. Science.

[28] Kleinstiver B.P., Prew M.S., Tsai S.Q., Topkar V.V., Nguyen N.T., Zheng Z., Gonzales A.P., Li Z., Peterson R.T., Yeh J.R. (2015). Engineered crispr-cas9 nucleases with altered pam specificities. Nature.

[29] Hu J.H., Miller S.M., Geurts M.H., Tang W., Chen L., Sun N., Zeina C.M., Gao X., Rees H.A., Lin Z. (2018). Evolved cas9 variants with broad pam compatibility and high DNA specificity. Nature.

[30] Walton R.T., Christie K.A., Whittaker M.N., Kleinstiver B.P. (2020). Unconstrained genome targeting with near-pamless engineered crispr-cas9 variants. Science.

[31] Cradick T.J., Fine E.J., Antico C.J., Bao G. (2013). Crispr/cas9 systems targeting beta-globin and ccr5 genes have substantial off-target activity. Nucleic Acids Res..

[32] Kim S., Kim D., Cho S.W., Kim J., Kim J.S. (2014). Highly efficient rna-guided genome editing in human cells via delivery of purified cas9 ribonucleoproteins. Genome Res..

[33] Rees H.A., Komor A.C., Yeh W.H., Caetano-Lopes J., Warman M., Edge A.S.B., Liu D.R. (2017). Improving the DNA specificity and applicability of base editing through protein engineering and protein delivery. Nat. Commun..

[34] Hsu P.D., Scott D.A., Weinstein J.A., Ran F.A., Konermann S., Agarwala V., Li Y., Fine E.J., Wu X., Shalem O. (2013). DNA targeting specificity of rna-guided cas9 nucleases. Nat. Biotechnol..

[35] Shalem O., Sanjana N.E., Hartenian E., Shi X., Scott D.A., Mikkelson T., Heckl D., Ebert B.L., Root D.E., Doench J.G. (2014). Genome-scale crispr-cas9 knockout screening in human cells. Science.

[36] Nelson C.E., Wu Y., Gemberling M.P., Oliver M.L., Waller M.A., Bohning J.D., Robinson-Hamm J.N., Bulaklak K., Castellanos Rivera R.M., Collier J.H. (2019). Long-term evaluation of aav-crispr genome editing for duchenne muscular dystrophy. Nat. Med..

[37] Ramakrishna S., Kwaku Dad A.B., Beloor J., Gopalappa R., Lee S.K., Kim H. (2014). Gene disruption by cell-penetrating peptide-mediated delivery of cas9 protein and guide rna. Genome Res..

[38] Zuris J.A., Thompson D.B., Shu Y., Guilinger J.P., Bessen J.L., Hu J.H., Maeder M.L., Joung J.K., Chen Z.Y., Liu D.R. (2015). Cationic lipid-mediated delivery of proteins enables efficient protein-based genome editing *in vitro* and *in vivo*. Nat. Biotechnol..

[39] Mout R., Ray M., Yesilbag Tonga G., Lee Y.W., Tay T., Sasaki K., Rotello V.M. (2017). Direct cytosolic delivery of crispr/cas9-ribonucleoprotein for efficient gene editing. Acs Nano.

[40] Mellott A.J., Forrest M.L., Detamore M.S. (2013). Physical non-viral gene delivery methods for tissue engineering. Ann. Biomed. Eng..

[41] Roos W.H., Ivanovska I.L., Evilevitch A., Wuite G.J.L. (2007). Viral capsids: Mechanical characteristics, genome packaging and delivery mechanisms. Cell Mol. Life Sci..

[42] Lidmar J., Mirny L., Nelson D.R. (2003). Virus shapes and buckling transitions in spherical shells. Phys. Rev. E Stat. Nonlinear Soft Matter Phys..

[43] Vernizzi G., Olvera de la Cruz M. (2007). Faceting ionic shells into icosahedra via electrostatics. Proc. Natl. Acad. Sci. USA.

[44] Durand S., Cimarelli A. (2011). The inside out of lentiviral vectors. Viruses.

[45] Mock U., Riecken K., Berdien B., Qasim W., Chan E., Cathomen T., Fehse B. (2014). Novel lentiviral vectors with mutated reverse transcriptase for mrna delivery of tale nucleases. Sci. Rep..

[46] Prel A., Caval V., Gayon R., Ravassard P., Duthoit C., Payen E., Maouche-Chretien L., Creneguy A., Nguyen T.H., Martin N. (2015). Highly efficient *in vitro* and *in vivo* delivery of functional rnas using new versatile ms2-chimeric retrovirus-like particles. Mol. Methods Clin. Dev..

[47] Knopp Y., Geis F.K., Heckl D., Horn S., Neumann T., Kuehle J., Meyer J., Fehse B., Baum C., Morgan M. (2018). Transient retrovirus-based crispr/cas9 all-in-one particles for efficient, targeted gene knockout. Mol. Ther. Nucleic Acids.

[48] Lu B., Javidi-Parsijani P., Makani V., Mehraein-Ghomi F., Sarhan W.M., Sun D., Yoo K.W., Atala Z.P., Lyu P., Atala A. (2019). Delivering sacas9 mrna by lentivirus-like bionanoparticles for transient expression and efficient genome editing. Nucleic Acids Res..

[49] Lindel F., Dodt C.R., Weidner N., Noll M., Bergemann F., Behrendt R., Fischer S., Dietrich J., Cartellieri M., Hamann M.V. (2019). Trafo-crispr: Enhanced genome engineering by transient foamy virus vector-mediated delivery of crispr/cas9 components. Mol. Ther. Nucleic Acids.

[50] Cai Y., Bak R.O., Mikkelsen J.G. (2014). Targeted genome editing by lentiviral protein transduction of zinc-finger and tal-effector nucleases. Elife.

[51] Choi J.G., Dang Y., Abraham S., Ma H., Zhang J., Guo H., Cai Y., Mikkelsen J.G., Wu H., Shankar P. (2016). Lentivirus pre-packed with cas9 protein for safer gene editing. Gene Ther..

[52] Mangeot P.E., Risson V., Fusil F., Marnef A., Laurent E., Blin J., Mournetas V., Massourides E., Sohier T.J.M., Corbin A. (2019). Genome editing in primary cells and *in vivo* using viral-derived nanoblades loaded with cas9-sgrna ribonucleoproteins. Nat. Commun..

[53] Gee P., Lung M.S.Y., Okuzaki Y., Sasakawa N., Iguchi T., Makita Y., Hozumi H., Miura Y., Yang L.F., Iwasaki M. (2020). Extracellular nanovesicles for packaging of crispr-cas9 protein and sgrna to induce therapeutic exon skipping. Nat. Commun..

[54] Indikova I., Indik S. (2020). Highly efficient ’hit-and-run’ genome editing with unconcentrated lentivectors carrying vpr.Prot.Cas9 protein produced from rre-containing transcripts. Nucleic Acids Res..

[55] Lyu P., Javidi-Parsijani P., Atala A., Lu B. (2019). Delivering cas9/sgrna ribonucleoprotein (rnp) by lentiviral capsid-based bionanoparticles for efficient ’hit-and-run’ genome editing. Nucleic Acids Res..

[56] Lu Z., Yao X., Lyu P., Yadav M., Yoo K., Atala A., Lu B. (2021). Lentiviral capsid-mediated spcas9 ribonucleoprotein delivery for efficient and safe multiplex genome editing. Cris. J..

[57] Lyu P., Lu Z., Cho S.I., Yadav M., Yoo K., Atala A., Kim J.S., Lu B. (2021). Adenine base editor ribonucleoproteins delivered by lentivirus-like particles show high on-target base editing and undetectable rna off-target activities. Cris. J..

[58] Payne S., Payne S. (2017). Chapter 36-family retroviridae. Viruses.

[59] Lingappa J.R., Reed J.C., Tanaka M., Chutiraka K., Robinson B.A. (2014). How hiv-1 gag assembles in cells: Putting together pieces of the puzzle. Virus Res..

[60] Briggs J.A., Simon M.N., Gross I., Krausslich H.G., Fuller S.D., Vogt V.M., Johnson M.C. (2004). The stoichiometry of gag protein in hiv-1. Nat. Struct. Mol. Biol..

[61] Cockrell A.S., Kafri T. (2007). Gene delivery by lentivirus vectors. Mol. Biotechnol..

[62] Parolin C., Sodroski J. (1995). A defective hiv-1 vector for gene transfer to human lymphocytes. J. Mol. Med. (Berl.).

[63] Kartikeyan S., Bharmal R.N., Tiwari R.P., Bisen P.S. (2007). Hiv and Aids: Basic Elements and Priorities.

[64] Spearman P., Wang J.J., Vander Heyden N., Ratner L. (1994). Identification of human immunodeficiency virus type 1 gag protein domains essential to membrane binding and particle assembly. J. Virol..

[65] Ganser-Pornillos B.K., von Schwedler U.K., Stray K.M., Aiken C., Sundquist W.I. (2004). Assembly properties of the human immunodeficiency virus type 1 ca protein. J. Virol..

[66] De Guzman R.N., Wu Z.R., Stalling C.C., Pappalardo L., Borer P.N., Summers M.F. (1998). Structure of the hiv-1 nucleocapsid protein bound to the sl3 psi-rna recognition element. Science.

[67] Freed E.O. (1998). Hiv-1 gag proteins: Diverse functions in the virus life cycle. Virology.

[68] Lu K., Heng X., Summers M.F. (2011). Structural determinants and mechanism of hiv-1 genome packaging. J. Mol. Biol..

[69] Grigorov B., Decimo D., Smagulova F., Pechoux C., Mougel M., Muriaux D., Darlix J.L. (2007). Intracellular hiv-1 gag localization is impaired by mutations in the nucleocapsid zinc fingers. Retrovirology.

[70] Hoshikawa N., Kojima A., Yasuda A., Takayashiki E., Masuko S., Chiba J., Sata T., Kurata T. (1991). Role of the gag and pol genes of human immunodeficiency virus in the morphogenesis and maturation of retrovirus-like particles expressed by recombinant vaccinia virus: An ultrastructural study. J. Gen. Virol..

[71] Akkina R.K., Walton R.M., Chen M.L., Li Q.X., Planelles V., Chen I.S. (1996). High-efficiency gene transfer into cd34+ cells with a human immunodeficiency virus type 1-based retroviral vector pseudotyped with vesicular stomatitis virus envelope glycoprotein g. J. Virol..

[72] Naldini L., Blomer U., Gallay P., Ory D., Mulligan R., Gage F.H., Verma I.M., Trono D. (1996). *In vivo* gene delivery and stable transduction of nondividing cells by a lentiviral vector. Science.

[73] Reiser J., Harmison G., Kluepfel-Stahl S., Brady R.O., Karlsson S., Schubert M. (1996). Transduction of nondividing cells using pseudotyped defective high-titer hiv type 1 particles. Proc. Natl. Acad. Sci. USA.

[74] Mattei S., Flemming A., Anders-Osswein M., Krausslich H.G., Briggs J.A., Muller B. (2015). Rna and nucleocapsid are dispensable for mature hiv-1 capsid assembly. J. Virol..

[75] Ortinski P.I., O’Donovan B., Dong X., Kantor B. (2017). Integrase-deficient lentiviral vector as an all-in-one platform for highly efficient crispr/cas9-mediated gene editing. Mol. Ther. Methods Clin. Dev..

[76] Hu J., Schokrpur S., Archang M., Hermann K., Sharrow A.C., Khanna P., Novak J., Signoretti S., Bhatt R.S., Knudsen B.S. (2018). A non-integrating lentiviral approach overcomes cas9-induced immune rejection to establish an immunocompetent metastatic renal cancer model. Mol. Ther. Methods Clin. Dev..

[77] Luis A. (2020). The old and the new: Prospects for non-integrating lentiviral vector technology. Viruses.

[78] Fouts D.E., True H.L., Celander D.W. (1997). Functional recognition of fragmented operator sites by r17/ms2 coat protein, a translational repressor. Nucleic Acids Res..

[79] Bertrand E., Chartrand P., Schaefer M., Shenoy S.M., Singer R.H., Long R.M. (1998). Localization of ash1 mrna particles in living yeast. Mol. Cell.

[80] Wu B., Chao J.A., Singer R.H. (2012). Fluorescence fluctuation spectroscopy enables quantitative imaging of single mrnas in living cells. Biophys. J..

[81] Ma H., Tu L.C., Naseri A., Huisman M., Zhang S., Grunwald D., Pederson T. (2016). Multiplexed labeling of genomic loci with dcas9 and engineered sgrnas using crisprainbow. Nat. Biotechnol..

[82] Zalatan J.G., Lee M.E., Almeida R., Gilbert L.A., Whitehead E.H., La Russa M., Tsai J.C., Weissman J.S., Dueber J.E., Qi L.S. (2015). Engineering complex synthetic transcriptional programs with crispr rna scaffolds. Cell.

[83] Muriaux D., Costes S., Nagashima K., Mirro J., Cho E., Lockett S., Rein A. (2004). Role of murine leukemia virus nucleocapsid protein in virus assembly. J. Virol..

[84] Holtkamp S., Kreiter S., Selmi A., Simon P., Koslowski M., Huber C., Tureci O., Sahin U. (2006). Modification of antigen-encoding rna increases stability, translational efficacy, and t-cell stimulatory capacity of dendritic cells. Blood.

[85] Schrom E., Huber M., Aneja M., Dohmen C., Emrich D., Geiger J., Hasenpusch G., Herrmann-Janson A., Kretzschmann V., Mykhailyk O. (2017). Translation of angiotensin-converting enzyme 2 upon liver- and lung-targeted delivery of optimized chemically modified mrna. Mol. Ther. Nucleic Acids.

[86] Hamann M.V., Stanke N., Müllers E., Stirnnagel K., Hütter S., Artegiani B., Bragado Alonso S., Calegari F., Lindemann D. (2014). Efficient transient genetic manipulation *in vitro* and *in vivo* by prototype foamy virus-mediated nonviral rna transfer. Mol. Ther. J. Am. Soc. Gene Ther..

[87] Hamann M.V., Müllers E., Reh J., Stanke N., Effantin G., Weissenhorn W., Lindemann D. (2014). The cooperative function of arginine residues in the prototype foamy virus gag c-terminus mediates viral and cellular rna encapsidation. Retrovirology.

[88] Ma H., Tu L.C., Naseri A., Huisman M., Zhang S., Grunwald D., Pederson T. (2016). Crispr-cas9 nuclear dynamics and target recognition in living cells. J. Cell Biol..

[89] Kaczmarczyk S.J., Sitaraman K., Young H.A., Hughes S.H., Chatterjee D.K. (2011). Protein delivery using engineered virus-like particles. Proc. Natl. Acad. Sci. USA.

[90] Peretti S., Schiavoni I., Pugliese K., Federico M. (2005). Cell death induced by the herpes simplex virus-1 thymidine kinase delivered by human immunodeficiency virus-1-based virus-like particles. Mol. Ther..

[91] Joo K.I., Wang P. (2008). Visualization of targeted transduction by engineered lentiviral vectors. Gene Ther..

[92] Vindry C., Guillin O., Mangeot P.E., Ohlmann T., Chavatte L. (2019). A versatile strategy to reduce uga-selenocysteine recoding efficiency of the ribosome using crispr-cas9-viral-like-particles targeting selenocysteine-trna([ser]sec) gene. Cells.

[93] Brown E.J., Albers M.W., Shin T.B., Ichikawa K., Keith C.T., Lane W.S., Schreiber S.L. (1994). A mammalian protein targeted by g1-arresting rapamycin-receptor complex. Nature.

[94] Sabatini D.M., Erdjument-Bromage H., Lui M., Tempst P., Snyder S.H. (1994). Raft1: A mammalian protein that binds to fkbp12 in a rapamycin-dependent fashion and is homologous to yeast tors. Cell.

[95] Gonzalez M.E. (2017). The hiv-1 vpr protein: A multifaceted target for therapeutic intervention. Int. J. Mol. Sci..

[96] Muller B., Tessmer U., Schubert U., Krausslich H.G. (2000). Human immunodeficiency virus type 1 vpr protein is incorporated into the virion in significantly smaller amounts than gag and is phosphorylated in infected cells. J. Virol..

[97] Selig L., Pages J.C., Tanchou V., Preveral S., Berlioz-Torrent C., Liu L.X., Erdtmann L., Darlix J., Benarous R., Benichou S. (1999). Interaction with the p6 domain of the gag precursor mediates incorporation into virions of vpr and vpx proteins from primate lentiviruses. J. Virol..

[98] Mukerjee R., Chang J.R., Del Valle L., Bagashev A., Gayed M.M., Lyde R.B., Hawkins B.J., Brailoiu E., Cohen E., Power C. (2011). Deregulation of micrornas by hiv-1 vpr protein leads to the development of neurocognitive disorders. J. Biol. Chem..

[99] Lyu P., Yoo K.W., Yadav M.K., Atala A., Aartsma-Rus A., Putten M.V., Duan D., Lu B. (2020). Sensitive and reliable evaluation of single-cut sgrnas to restore dystrophin by a gfp-reporter assay. PLoS ONE.

[100] Lim F., Downey T.P., Peabody D.S. (2001). Translational repression and specific rna binding by the coat protein of the pseudomonas phage pp7. J. Biol. Chem..

[101] Austin R.J., Xia T., Ren J., Takahashi T.T., Roberts R.W. (2002). Designed arginine-rich rna-binding peptides with picomolar affinity. J. Am. Chem. Soc..

[102] Wulczyn F.G., Kahmann R. (1991). Translational stimulation: Rna sequence and structure requirements for binding of com protein. Cell.

[103] Grunewald J., Zhou R., Garcia S.P., Iyer S., Lareau C.A., Aryee M.J., Joung J.K. (2019). Transcriptome-wide off-target rna editing induced by crispr-guided DNA base editors. Nature.

[104] Zhou C., Sun Y., Yan R., Liu Y., Zuo E., Gu C., Han L., Wei Y., Hu X., Zeng R. (2019). Off-target rna mutation induced by DNA base editing and its elimination by mutagenesis. Nature.

[105] Rees H.A., Wilson C., Doman J.L., Liu D.R. (2019). Analysis and minimization of cellular rna editing by DNA adenine base editors. Sci. Adv..

[106] Farboud B., Jarvis E., Roth T.L., Shin J., Corn J.E., Marson A., Meyer B.J., Patel N.H., Hochstrasser M.L. (2018). Enhanced genome editing with cas9 ribonucleoprotein in diverse cells and organisms. J. Vis. Exp..

[107] Hinz J.M., Laughery M.F., Wyrick J.J. (2015). Nucleosomes inhibit cas9 endonuclease activity *in vitro*. Biochemistry.

[108] Horlbeck M.A., Witkowsky L.B., Guglielmi B., Replogle J.M., Gilbert L.A., Villalta J.E., Torigoe S.E., Tjian R., Weissman J.S. (2016). Nucleosomes impede cas9 access to DNA *in vivo* and *in vitro*. Elife.

[109] Isaac R.S., Jiang F., Doudna J.A., Lim W.A., Narlikar G.J., Almeida R. (2016). Nucleosome breathing and remodeling constrain crispr-cas9 function. Elife.

[110] Kallimasioti-Pazi E.M., Thelakkad Chathoth K., Taylor G.C., Meynert A., Ballinger T., Kelder M.J.E., Lalevee S., Sanli I., Feil R., Wood A.J. (2018). Heterochromatin delays crispr-cas9 mutagenesis but does not influence the outcome of mutagenic DNA repair. Plos Biol..

[111] DePolo N.J., Reed J.D., Sheridan P.L., Townsend K., Sauter S.L., Jolly D.J., Dubensky T.W. (2000). Vsv-g pseudotyped lentiviral vector particles produced in human cells are inactivated by human serum. Mol. Ther..

[112] Brown B.D., Sitia G., Annoni A., Hauben E., Sergi L.S., Zingale A., Roncarolo M.G., Guidotti L.G., Naldini L. (2007). *In vivo* administration of lentiviral vectors triggers a type i interferon response that restricts hepatocyte gene transfer and promotes vector clearance. Blood.

[113] Schauber-Plewa C., Simmons A., Tuerk M.J., Pacheco C.D., Veres G. (2005). Complement regulatory proteins are incorporated into lentiviral vectors and protect particles against complement inactivation. Gene Ther..

[114] Milani M., Annoni A., Moalli F., Liu T., Cesana D., Calabria A., Bartolaccini S., Biffi M., Russo F., Visigalli I. (2019). Phagocytosis-shielded lentiviral vectors improve liver gene therapy in nonhuman primates. Sci. Transl. Med..

[115] Milani M., Annoni A., Bartolaccini S., Biffi M., Russo F., Di Tomaso T., Raimondi A., Lengler J., Holmes M.C., Scheiflinger F. (2017). Genome editing for scalable production of alloantigen-free lentiviral vectors for *in vivo* gene therapy. Embo Mol. Med..

